# “Look at me, not your screen”: the negative association of parental phubbing on adolescent well-being

**DOI:** 10.3389/fpsyt.2026.1869469

**Published:** 2026-07-21

**Authors:** Qi Liu, Qi Cui, Myduyen Dang, Jihe Chen, Bing Shi

**Affiliations:** 1 School of Teacher Development, Shaanxi Normal University, Xi’an, Shaanxi, China; 2 School of Education, Shanghai Jiao Tong University, Shanghai, China; 3 The Faculty of Chinese Language and Culture, Dong A University, Danang, Vietnam; 4 The Faculty of Education, Southwest University, Beibei, Chongqing, China; 5 Physical Education School, Shaanxi Normal University, Xi’an, Shaanxi, China

**Keywords:** adolescent well-being, generalized anxiety, parental phubbing, physical activity, social anxiety

## Abstract

Against the backdrop of rapid digital technology development, the impact of parental phubbing on adolescents’ physical and mental health has garnered increasing attention. Grounded in the Stimulus-Organism-Response (SOR) theoretical model, this study examines the statistical associations linking parental phubbing to adolescents’ anxiety levels and participation in physical activity. Data were collected from 550 middle school students using the Parental Phubbing Scale (PPS), the Generalized Anxiety Disorder-7 (GAD-7), the Social Anxiety Disorder Scale-7 (SAD-7), and the Physical Activity Rating Scale-3 (PARS-3). After excluding 41 invalid responses, 509 valid questionnaires were included in the final analysis. The findings reveal that frequent parental phubbing is significantly positively associated with adolescents’ generalized anxiety and social anxiety, and negatively associated with their engagement in physical activity. Network analysis further shows that generalized anxiety emerges as a central hub in the network, whereas social anxiety exhibits a distinct suppressive moderating effect. These results highlight the potential harm of shifting family interaction patterns in the digital age and emphasize the need for parents to be mindful of the modeling effects of their device use. Strengthening face-to-face interactions may help promote adolescents’ physical and psychological well-being. These findings offer correlational evidence that may inform the establishment of family digital behavior guidelines and the development of intervention strategies to support adolescent health.

## 1 Introduction

In the digital era, family life is increasingly permeated by smartphones and other electronic devices. These technologies provide convenient communication, abundant entertainment, and efficient information access (1, 2). However, their widespread adoption has also introduced significant concerns. Parents’ frequent use of mobile devices (parental phubbing) while spending time with adolescents, has emerged as a growing issue in both academic and public discourse. This behavior not only diminishes the quality of parent-child interactions but may also exert profound effects on adolescents’ mental health and behavioral patterns, being associated with negative emotions such as anger and sadness (2, 3). Parents serve as crucial role models for adolescents, and when device use intrudes upon parent-child interactions, disregarding adolescents’ emotional or behavioral needs, it is statistically associated with both parental problematic smartphone dependency and relational neglect (4). Notably, parental phubbing extends beyond the home, occurring in contexts such as family meals outings and shared activities (5). Chronic engagement in this behavior may erode parent-child bonds while adversely affecting children’s emotional regulation, cognitive development, and long-term psychological and physical health (3, 6).

Another topic associated with the prevalence of smartphones is the declining trend in adolescents’ mental health and physical well-being (7). Current research primarily focuses on the relationship between parental phubbing and adolescents’ smartphone addiction (8), academic burnout (9), and depressive tendencies (10). These studies suggest that parental phubbing is statistically associated with poorer mental health outcomes in adolescents, increasing the likelihood of emotional disorders, diminished social skills, and identity crises (11).

However, among the various manifestations of adolescents’ mental health, generalized anxiety (GAD) and social anxiety (SAD) have received less attention compared to depression or smartphone addiction. Unlike depression which is a broader affective disorder influenced by multiple genetic and environmental factors, GAD is more directly linked to a lack of security and predictability in daily parent child interactions (12). Unlike smartphone addiction which may share behavioral patterns with parental phubbing, SAD is more directly related to reduced opportunities for face-to-face social learning and heightened fear of negative evaluation (13). Specifically, GAD is characterized by excessive worry about daily life events and may stem from a lack of security caused by insufficient parent child interaction (14). SAD is reflected in interpersonal tension and avoidance and is directly linked to a lack of effective face-to-face communication. Therefore, focusing on GAD and SAD allows us to capture the immediate emotional consequences of parental phubbing more precisely than broader outcomes like depression or behaviorally oriented outcomes like smartphone addiction. The emergence of these two types of anxiety causes numerous challenges in adolescents’ academic lives, social interactions, and other areas.

Beyond these direct associations, parental phubbing may also indirectly influence adolescents’ physical activity through psychological pathways. Two candidate mechanisms are particularly worth considering. First, parental phubbing could reduce adolescents’ self-efficacy in sports (15). Parents serve as primary role models and sources of encouragement for physical activity (16). When parents are absorbed in their devices, they provide less verbal encouragement and fewer opportunities for joint exercise. This may lead adolescents to doubt their own athletic competence. Second, parental phubbing may impair adolescents’ emotion regulation abilities. Frequent interruptions in parent child communication deprive adolescents of consistent emotional support and modeling of appropriate coping strategies. Poor emotion regulation could then make adolescents more vulnerable to anxiety, which in turn discourages physical activity.

The Stimulus Organism Response (SOR) theoretical model provides an effective analytical framework for studying this complex issue (17, 18). This model describes a theoretical process by which external stimuli relate to an individual’s internal psychological state, which in turn relates to specific behavioral responses (19). This study adopts the SOR model to explore the intrinsic connections among parental phubbing, adolescents’ mental health, and their physical activity patterns. This research not only helps uncover the mechanisms through which parental behavior in the family environment affects adolescents’ psychological and behavioral outcomes but also provides theoretical support and practical guidance for improving parent child relationships and promoting adolescents’ mental well-being.

## 2 Literature review

### 2.1 Definition and impact of parental phubbing

Parental phubbing is fundamentally a form of social exclusion behavior that can exert profound negative effects on interpersonal relationships. In this study, we define “parental phubbing” as a phenomenon in which parents use their mobile devices in a way that makes their child feel excluded during parent-child interactions (20). This definition emphasizes two key characteristics: the situational context, occurring during parent-child interactions and the negative nature, unwarranted usage. Such an operational definition helps us observe and measure this phenomenon more accurately, providing a clear conceptual framework for subsequent research. Notably, this definition excludes parents’ necessary phone use for legitimate reasons (e.g., work) and specifically refers to non-essential mobile use that disrupts the quality of parent-child interactions. This behavior has become increasingly prevalent in the digital age, with its core feature being parents diverting attention from face-to-face interactions to electronic devices, thereby interrupting emotional communication (21).

Existing research demonstrates that parental phubbing exerts profound impacts on children’s and adolescents’ psychological development (22). From a social psychological perspective, phubbing constitutes a subtle yet significant form of social exclusion (23). When parents frequently use their phones during parent-child interactions, they inadvertently send their children the message that “devices are more important than interpersonal relationships.” This signal can damage parent-child attachment bonds, diminish adolescents’ sense of security, and lower their self-worth. A longitudinal study found that children who experience long-term parental phubbing are more prone to emotional regulation difficulties and deficits in social skills (11). Notably, these effects may be particularly pronounced during adolescence, as this developmental stage is characterized by heightened sensitivity to social feedback (24).

### 2.2 Types of adolescent anxiety and related factors

In the field of adolescent mental health, generalized anxiety and social anxiety are recognized as two of the most representative types of anxiety disorders (25). The prevalence of these anxiety forms among adolescents has been steadily rising, emerging as a global public health concern (26). From a developmental psychology perspective, family environmental factors play a crucial role in the formation and maintenance of adolescent anxiety. Specifically, parental behavior patterns and parenting styles profoundly influence adolescents’ emotion regulation abilities through mechanisms such as observational learning and emotional transmission (27). Anxiety, as a category of emotion, involves dynamic bidirectional influences between parents and children, where emotional changes in one can affect the other (28). Since adolescence is a critical period for social skill development, parents’ distracted attention during interactions may limit adolescents’ opportunities to observe and learn appropriate social behaviors. This, in turn, can amplify feelings of uncertainty and anxiety in social situations (29).

It is important to distinguish GAD and SAD from other commonly studied outcomes in parental phubbing research such as depression and smartphone addiction. Depression tends to develop over longer periods and is influenced by cumulative risks like peer victimization or genetic vulnerability. In contrast GAD and SAD can arise more immediately from perceived parental neglect and social exclusion during daily interactions. Smartphone addiction is a behavioral outcome that may reflect imitation or habit formation. In contrast GAD and SAD represent internalizing emotional states that are theoretically closer to the “Organism” component of the SOR model. This makes them more appropriate mediators between parental phubbing as a stimulus and physical activity avoidance as a response. This distinction constitutes a key innovation of the present study.

### 2.3 Causes and effects of sports avoidance

Youth and sport represent a critical issue in the global pursuit of sustainable educational development, directly contributing to the achievement of the Sustainable Development Goals (SDGs), particularly in health (SDG 3), education (SDG 4), gender equality (SDG 5), and social inclusion (SDG 10) (30). Participation in sports activities offers multifaceted benefits for the physical and mental health development of adolescents, including enhanced physical fitness, improved emotional well-being, and the cultivation of social skills (31). Research by Strandbu et al. indicates that parental behavior patterns significantly influence adolescents’ attitudes and engagement in sports activities. Parents shape their children’s active lifestyles through various means, such as role modeling, sports initiation, participation encouragement, resource support, and expressions of interest in sports (32). However, when parents exhibit excessive use of electronic devices during family interactions, it not only reduces their own participation in sports activities but may also reinforce their children’s preference for sedentary lifestyles through observational learning. Further studies reveal that parental phubbing behavior may indirectly diminish adolescents’ willingness to engage in sports by triggering smartphone addiction (33).

### 2.4 SOR model

The SOR model provides a robust theoretical framework for understanding the mechanisms underlying the effects of parental phubbing behavior. This model emphasizes that external environmental stimuli influence individuals’ internal psychological states, thereby triggering specific behavioral responses (19, 34). The strength of this theoretical framework lies in its ability to systematically reveal causal relationships between variables, particularly the mediating role of internal psychological states (35). Specifically, this study hypothesizes that parental phubbing, as a chronic stressor, exacerbates adolescents’ anxiety by undermining their sense of security and social connectedness, thereby fostering a behavioral pattern of avoiding physical activity. This theoretical pathway not only aligns with contemporary developmental psychology’s understanding of environmental influence mechanisms but also corresponds with clinical observations highlighting the strong association between anxiety and behavioral avoidance. By applying the SOR model, this study aims to provide a more systematic explanation of the long-term effects of parental phubbing behavior.

## 3 Research hypotheses

### 3.1 Parental phubbing and adolescent mental health

When parents frequently use their phones during interactions with adolescents, this distracted behavior sends signals of emotional neglect and undermines the quality of parent-child communication (6). The “Digital Diary” research project by AVG, which conducted in-depth interviews with 6, 000 families (parents and their children aged 8-13), revealed concerning findings. The data showed that 32% of the surveyed children felt “unimportant” due to their parents’ phone addiction, while more than half (54%) believed their parents used mobile devices excessively (36). Specifically, parental phubbing reduces meaningful emotional exchanges and interaction time, depriving adolescents of adequate emotional support and a sense of security. This deprivation can lead to excessive worry about daily life events, contributing to generalized anxiety. Additionally, as primary social role models, parents’ phubbing behavior limits adolescents’ opportunities to observe and learn appropriate social skills. Adolescents may internalize this behavior as a cognitive distortion that social interactions are unimportant, thereby experiencing heightened social anxiety in real-life social situations. Based on this, the study proposes the following hypotheses,

H1a: Parental phubbing will be significantly and positively associated with adolescents’ generalized anxiety.
H1b: Parental phubbing will be significantly and positively associated with adolescents’ social anxiety.

### 3.2 Parental phubbing and adolescents’ avoidance of physical activity

Based on family systems theory, many researchers suggest that parental behavioral patterns influence adolescents’ participation in physical activity through various pathways. Parental phubbing not only directly affects their children’s family-based physical activity but also indirectly restricts it by influencing their children’s phubbing behavior (37). This is reflected in the way parental phubbing directly reduces the time and opportunities for parent-child joint participation in physical activity (11). Specifically, parental phubbing leads to perceived neglect, a lack of emotional connection, and reduced communication between parents and adolescents (38). As a result, adolescents may experience elevated stress, diminished self-esteem, and difficulties in forming secure attachment with their parents.

At the same time, parents’ excessive use of mobile phones sets an example of a sedentary lifestyle for adolescents, diminishing their value recognition of physical activity. Additionally, parents’ phubbing behavior while accompanying adolescents in sports carries the hidden risk of weakening the adolescents’ experience of enjoyment and sense of achievement in physical activity. Studies indicate that parental attention and encouragement are important sources of adolescents’ sense of achievement in sports (39), and phubbing significantly diminishes this social reinforcement, making children feel their efforts are unappreciated, thereby reducing their motivation to engage in physical activity. This study hypothesizes,

H2: Parental phubbing will be significantly and negatively associated with adolescents’ level of physical activity.

### 3.3 Adolescent mental health and avoidance of physical activity

There exists a complex interaction mechanism between adolescents’ mental health status and their participation in physical activity. From a cognitive-behavioral perspective, children with generalized anxiety traits often exhibit hypervigilance toward bodily sensations during physical activity (40). They tend to catastrophize normal physiological responses—such as increased heart rate and shortness of breath during exercise—interpreting them as danger signals. This cognitive bias significantly reduces their willingness to engage in physical activity. Anxious adolescents also display impairments in peer relationships; they are less popular, exhibit poorer social performance, are more frequently victimized, and have fewer friends compared to their non-anxious peers (41).

Meanwhile, children with social anxiety traits experience more intense distress from the “audience effect” during physical activity, as they excessively focus on others’ evaluations of them (42). Adolescents with higher levels of anxiety fear receiving negative evaluations when their athletic performance is subpar, and this apprehension leads to avoidance of team-based physical activities (43). Chronic anxiety alters children’s stress response systems, causing them to react more negatively to the physiological arousal induced by exercise (44). This vicious cycle of physiological and psychological reactions further reinforces their negative attitudes toward physical activity. This study hypothesizes,

H3a: Adolescents’ generalized anxiety will be significantly and negatively associated with their level of physical activity.
H3b: Adolescents’ social anxiety will be significantly and negatively associated with their level of physical activity.

### 3.4 The mediating role of mental health

**Figure 1:**
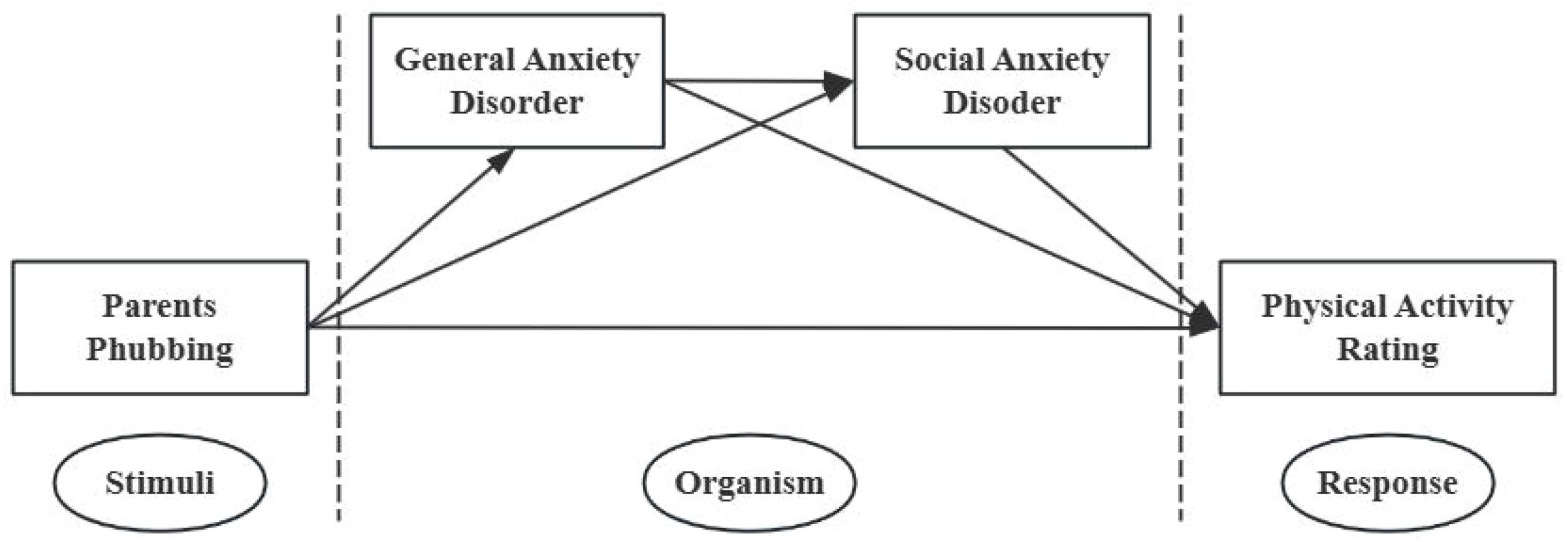
Research framework.

This hypothesis integrates the mechanisms of the three aforementioned hypotheses, constructing a complete “Stimulus-Organism-Response” (SOR) pathway (**Figure 1**). Parental phubbing, as a negative environmental stimulus, first impairs adolescents’ mental health (organism), manifesting as increased levels of generalized anxiety and social anxiety. This heightened anxiety then leads adolescents to adopt avoidance behaviors toward physical activity (response). This study hypothesizes,

H4a: Generalized anxiety plays a mediating role in the statistical association between parental phubbing and adolescents’ physical activity.
H4b: Social anxiety plays a mediating role in the statistical association between parental phubbing and adolescents’ physical activity.
H4c: Generalized anxiety and social anxiety play a chain mediation role in the statistical association between parental phubbing and adolescents’ physical activity

### 3.5 Research model based on SOR

In summary, the literature review above suggests three key theoretical associations that warrant empirical examination. First, parental phubbing may function as a notable environmental stimulus. It may signal emotional neglect and disrupt the quality of parent child communication. According to the SOR model, such external stimuli may be associated with changes in the individual’s internal psychological state. Second, generalized anxiety and social anxiety are proposed as the organism component of the model. Parental phubbing may be positively associated with both types of anxiety by undermining adolescents’ sense of security and limiting their opportunities for face-to-face social learning. Third, physical activity avoidance is proposed as the behavioral response. Heightened anxiety, particularly generalized anxiety and social anxiety, may be associated with adolescents’ withdrawal from physical activities due to fear of negative evaluation or physiological hyperarousal. The SOR model thus provides a coherent framework for exploring the statistical associations linking parental phubbing (stimulus) to anxiety (organism) and ultimately to physical activity avoidance (response). The following hypotheses and **Figure 1** formalize these proposed theoretical pathways.

## 4 Methods

### 4.1 Procedure

This study employs a combined approach of PLS-SEM and JASP network analysis to leverage the complementary strengths of both analytical techniques. First, PLS-SEM is used for model validation. This method can simultaneously handle complex measurement and structural models, ensuring measurement quality by assessing composite reliability (CR > 0.7), convergent validity (AVE > 0.5), and discriminant validity (HTMT < 0.85). Path coefficients are tested for statistical significance using the Bootstrap method, and the model’s explanatory power (R²) and predictive validity (Q²) are evaluated (45). Subsequently, network analysis is conducted using JASP. Its intuitive graphical interface and robust modeling capabilities allow for an in-depth exploration of complex correlational networks among variables. Automated centrality analysis and Bootstrap stability testing ensure the reliability of the network structure (29). The integration of these two methods serves complementary purposes: PLS-SEM tests the hypothesized SOR pathways, while network analysis identifies central symptom nodes and explores conditional associations among items. Due to the cross-sectional design, neither method can demonstrate dynamic or causal relationships.

### 4.2 Ethical approval and informed consent

This study employed a quantitative, non-experimental online survey. This study was approved by the Academic Committee of Shaanxi Normal University (Ethics Approval Number: 202616035). The study involved human participants and specifically included minors (middle school students).

Since data were collected via an online questionnaire platform (www.wjx.cn), written informed consent was obtained electronically. Before the questionnaire began, a consent statement was presented to the legal guardians of the minor participants. Guardians were required to check a box indicating “I have read and understood the study information and agree to my child’s participation.” Only after guardian consent was recorded could the minor proceed to the questionnaire. Minor participants were then presented with an assent statement and were required to check a box indicating “I agree to participate.” This online checkbox procedure served as the equivalent of written informed consent.

As this was a non-experimental cross-sectional survey, no experimental manipulation, random assignment, scenario priming, manipulation checks, or post-experiment debriefing were involved. All data were collected anonymously. No personal identifiers (e.g., names, student IDs, or home addresses) were recorded. IP addresses were not logged. All responses were stored on a password-protected computer accessible only to the research team. Data were analyzed only in de-identified, aggregated form.

All procedures performed in this study were in accordance with the ethical standards of the institutional research committee and with the 1964 Helsinki Declaration and its later amendments.

### 4.3 Measurement instrument

This study employed a cross-sectional questionnaire survey design. All measures were administered using validated self-report scales without experimental manipulation or scenario priming.

The respondents were middle school students who were asked to complete a questionnaire consisting of personal information and core research variables. Personal information included basic demographic details such as gender, grade level, and residential location. Core research variables were measured using validated scales, including parental phubbing behavior, student anxiety levels, and physical activity levels.

To ensure the validity of the data, this study employed Harman’s single-factor test to examine common method bias (CMV). The results showed that there were four factors with eigenvalues greater than 1, with the first factor explaining only 19.104% of the variance—significantly below the critical threshold of 40% (46, 47). This indicates that the self-reported data did not suffer from severe common method bias, supporting the reliability and validity of the study’s findings.

#### 4.3.1 Parental phubbing scale

Parental phubbing behavior was assessed using the Parental Phubbing Scale (PPS) revised by J. Li et al. (48). This scale consists of 9 items (e.g., “When I eat with my parents, they use their phones”). The scale has been validated in Chinese adolescent samples and demonstrated good psychometric properties. To ensure age-appropriateness for middle school students, the wording of the scale was reviewed by two experts in developmental psychology, who confirmed that all items were easily understandable for this age group. No further adaptation was deemed necessary. The questionnaire employs a 5-point Likert scale (1=never, 5=always), with higher scores indicating more severe phubbing behavior by parents during interactions with adolescents. In this study, the internal consistency coefficient of the scale was 0.894.

#### 4.3.2 Generalized anxiety disorder-7

Generalized anxiety was measured using the Generalized Anxiety Disorder-7 (GAD-7) scale (49). The Chinese version of the GAD-7 has been widely validated and used in adolescent populations. To maintain consistency with the other scales in this study (all of which adopted a 5-point Likert scale) and to facilitate ease of completion for adolescent participants, the standard four-option response format of the GAD-7 (scored 0–3) was adapted to a 5-point scale. The adapted response options were: “Not at all” (1), “Several days” (2), “More than half the days” (3), “Nearly every day” (4), and “Almost always” (5). This adaptation was reviewed by two experts to ensure that the meaning of the original items remained unchanged. Following this modification, the scale demonstrated satisfactory psychometric properties (Cronbach’s α = 0.869).

#### 4.3.3 Social anxiety disorder scale-7

Social anxiety was assessed using the Social Anxiety Disorder Scale-7 (SAD-7) (50). The Chinese version of the SAD-7 has been previously validated. To ensure comprehension for middle school students, minor wording adjustments were made based on their age characteristics. Specifically, a panel of three experts (two developmental psychologists and one middle school teacher) reviewed the original items and recommended specifying general terms (e.g., “social situations” was specified as “class activities or gatherings with classmates, ” which are common social scenarios for students). A pre-test was conducted with 30 middle school students, who confirmed that the adapted items were clear and easy to understand. No translation or back-translation was required, as the Chinese version was already validated and only minor wording adjustments were made without altering the core meaning of the items. Content validity was confirmed by the expert panel. The scale consists of 7 items scored on a 5-point Likert scale (1 = never, 5 = always), with higher scores indicating greater severity of social anxiety. In this study, the scale demonstrated a Cronbach’s alpha coefficient of 0.863.

#### 4.3.4 Physical activity rating scale-3

Physical activity was measured using the Physical Activity Rating Scale-3 (PARS-3) (51), which has been validated in Chinese adolescent samples. This scale evaluates physical activity levels based on three dimensions: intensity, duration, and frequency. To maintain consistency with the other scales in this study (all of which adopted a 5-point Likert scale), we converted the scoring of each dimension to a uniform 5-point scale scored from 1 to 5 (52). For intensity, the five levels were: “light exercise (e.g., walking, calisthenics)” (1), “low-intensity exercise (e.g., recreational volleyball, table tennis, jogging, Tai Chi)” (2), “moderate-intensity sustained exercise (e.g., cycling, running, table tennis)” (3), “high-intensity but non-sustained exercise causing heavy breathing and sweating (e.g., badminton, basketball, tennis, football)” (4), and “high-intensity sustained exercise causing heavy breathing and sweating (e.g., racing, complete aerobics routines, swimming)” (5). For duration, the five levels were: “less than 10 minutes” (1), “11–20 minutes” (2), “21–30 minutes” (3), “31–59 minutes” (4), and “60 minutes or more” (5). For frequency, the five levels were: “less than once a month” (1), “2–3 times per month” (2), “1–2 times per week” (3), “3–5 times per week” (4), and “approximately once per day” (5). A total physical activity score was calculated by summing the scores of the three dimensions. In this study, the scale demonstrated a Cronbach’s alpha coefficient of 0.767.

### 4.4 Sample source, recruitment, and data quality control

Participants were recruited using a stratified sampling method from three urban schools in Shaanxi Province, China. Stratification was based on grade level (grades 7, 8, and 9), and within each grade, one or two classes were randomly selected to ensure proportional representation. Inclusion criteria for participants were: currently enrolled in grades 7 to 9, having at least one parent or primary caregiver, and being able to understand and complete the questionnaire independently. The research team first contacted school administrators and obtained school approval. Electronic informed consent forms (via www.wjx.cn) were then distributed to parents or guardians through class WeChat groups managed by homeroom teachers. Participation was voluntary, and no incentives were provided. A total of 550 students initially completed the questionnaire. Among the invited participants, no students refused to participate after guardian consent was obtained, and all participating students provided guardian consent prior to data collection.

The online questionnaire (www.wjx.cn) was configured with several quality control features. All questions were set as required, meaning participants could not submit the questionnaire unless every item was answered; consequently, no missing data were present in the collected responses. Each IP address was permitted to submit the questionnaire only once, preventing duplicate submissions from the same device or network. A minimum response time threshold of 120 seconds (2 minutes) was set based on pilot testing, which indicated that careful reading and completion required at least 2 minutes; questionnaires completed in less than 120 seconds were excluded, though no responses fell below this threshold in the final sample. Responses were classified as invalid and excluded if participants selected the same answer option for all items (i.e., straightlining), and a total of 41 questionnaires were removed for this reason. No attention check items were included in this survey, as the forced-response format and straightlining detection were considered sufficient for identifying careless responding. After applying the above exclusion criteria, 509 valid questionnaires remained from the initial 550 responses, yielding an effective response rate of 92.55%.

### 4.5 Participants

**Table 1** presents the statistical overview of the sample’s demographic information, including gender, grade, family residence, and whether the respondent was an only child. Among the 509 samples, males accounted for 56.2%, while females made up 43.8%, indicating a relatively balanced gender distribution. In terms of grade composition, first-year middle school students constituted the largest proportion at 36.3%, followed by second-year students at 25.3%, and third-year students at 38.3%. The number of participants across grades was relatively evenly distributed.

**Table 1:** Participant information (N = 509).

Variable	Description	N	%
Gender	Male	286	56.2
	Female	223	43.8
Grade	7	185	36.3
	8	129	25.3
	9	195	38.3
Residence	Town	206	40.5
	Rural area	303	59.5
Only child or not	Yes	222	43.6
	No	287	56.4

## 5 Results

### 5.1 Reliability and validity tests

This study employed rigorous statistical methods to systematically examine the construct validity and reliability of the scales (**Table 2**). For validity testing, confirmatory factor analysis (CFA) was conducted to assess factor loadings. The results showed that the standardized factor loadings of all items ranged between 0.613 and 0.832 (all exceeding the acceptable threshold of 0.60), indicating good construct validity (53, 54). However, one item from the initial Parental Phubbing Scale with a factor loading below 0.5 was removed.

**Table 2:** Results of factor loading, C.R and AVE (N = 509).

Variable	Factor loading	C.R	AVE
Parental Phubbing	0.723-0.830	0.897	0.576
General Anxiety Disorder	0.613-0.815	0.876	0.564
Social Anxiety Disorder	0.627-0.812	0.861	0.551
Physical Activity Rating	0.820-0.832	0.769	0.681

For reliability and convergent validity testing, CFA further provided composite reliability (C.R.) and average variance extracted (AVE) values. The results showed that the C.R. values ranged from 0.769 to 0.897 (all above the 0.7 threshold), and the AVE values fell between 0.551 and 0.681 (all meeting the 0.5 standard), confirming that the scales exhibited satisfactory internal consistency and convergent validity (55).

### 5.2 Discriminant validity test

After examining the reliability and validity of the questionnaire, this study further investigated the discriminant validity among the factors. According to the Fornell-Larcker criterion, a scale demonstrates satisfactory discriminant validity when the square root of a factor’s average variance extracted (AVE) is greater than its Pearson correlation coefficients with other factors (53). As shown in **Table 3**, the values on the diagonal represent the square roots of the AVE for each factor (reflecting the strength of internal correlations within the factor), while the off-diagonal values indicate the Pearson correlation coefficients between factors. The analysis results revealed that the square roots of the AVE for all factors were significantly greater than their correlation coefficients with other factors, confirming that the questionnaire exhibits good discriminant validity.

**Table 3:** Results of discrimination test (N = 509).

Variable	GAD	PAR	PP	SAD
General Anxiety Disorder	**0.751**			
Physical Activity Rating	-0.414***	**0.825**		
Parental Phubbing	0.564***	-0.504***	**0.759**	
Social Anxiety Disorder	0.619***	-0.466***	0.649***	**0.742**

*p<0.1, **p<0.05, ***p<0.001.

As shown in **Table 4**, all HTMT values were below the recommended threshold of 0.85. These results support the discriminant validity among the four constructs.

**Table 4:** The result of HTMT.

Variable	GAD	PAR	PP	SAD
General Anxiety Disorder				
Physical Activity Rating	0.504			
Parental Phubbing	0.629	0.605		
Social Anxiety Disorder	0.692	0.563	0.732	

### 5.3 Structural model evaluation

**Figure 2:**
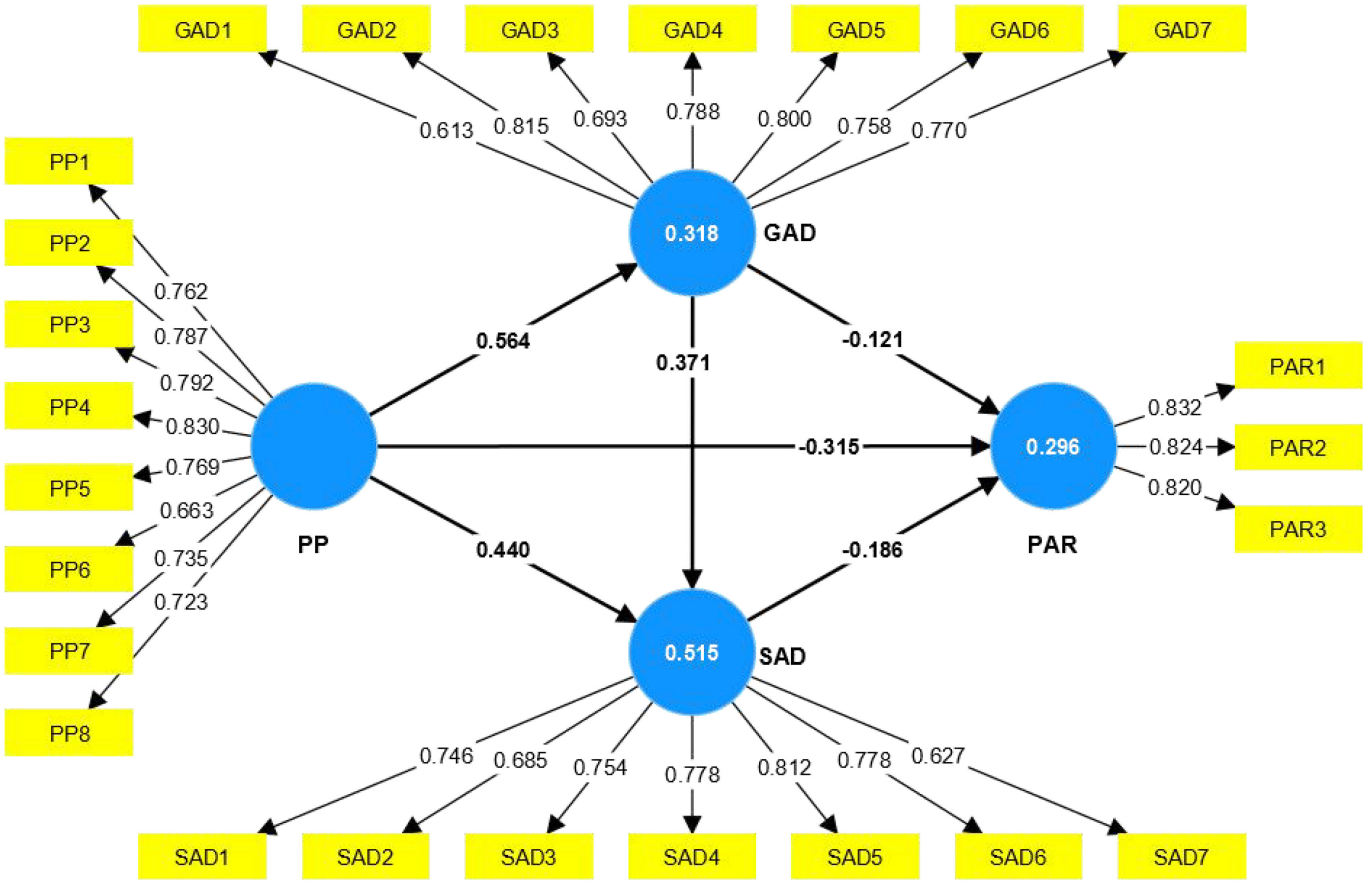
Path coefficient.

For effect size evaluation, the study adopted Hair et al.’ (56) criteria, classifying R² values of 0.75, 0.50, and 0.25 as representing substantial, moderate, and weak explanatory power, respectively. As illustrated in **Figure 2**, the R² values for the three core outcome variables were as follows: social anxiety (SAD) = 0.318, generalized anxiety (GAD) = 0.515, and physical activity (PAR) = 0.296.

According to the aforementioned benchmarks, the model demonstrates moderate explanatory power for generalized anxiety. For social anxiety and physical activity, the explanatory power falls between the weak and moderate range, indicating that while the model explains meaningful variance in these outcomes, a substantial portion of variance remains unaccounted for by the current set of predictors. This modest explanatory power is not unexpected given the multifactorial nature of adolescent anxiety and physical activity, which are influenced by additional factors not included in this model (e.g., peer relationships, school environment, genetic predispositions).

The overall model fit, measured by Standardized Root Mean Square Residual (SRMR), yielded a value of 0.076, which is below the commonly recommended threshold of 0.08, indicating acceptable model fit. Collectively, these findings provide preliminary empirical support for the proposed theoretical pathways (57).

**Table 5** presents the detailed test results of the nine research hypotheses in the model, including the standardized coefficients, T-statistics, and P-values for each path. All path coefficients and significance tests were estimated using the bootstrap method with 5, 000 resamples. According to the statistical significance criteria (T > 1.96 and P < 0.05), all nine initial hypotheses reached statistically significant levels (58). To further support the mediation path testing, 95% bias-corrected bootstrap confidence intervals (CIs) were calculated for all indirect effects. As shown in **Table 5**, none of the CIs for H4a, H4b, or H4c contained zero, indicating significant indirect associations.

**Table 5:** Inspection results of the path (N = 509).

Hypothesis	Path	β	T statistics	Upper CI	Lower CI	P value
H1a	PP→GAD	0.564	14.879	0.475	0.627	<0.000
H1b	PP→SAD	0.440	9.773	0.353	0.53	<0.000
H2	PP→PAR	-0.315	6.410	-0.413	-0.221	<0.000
H3a	GAD→ PAR	-0.121	2.216	-0.227	-0.015	0.027
H3b	SAD→PAR	-0.186	3.426	-0.292	-0.077	0.001
H4a	PP→GAD→PAR	-0.068	2.130	-0.133	-0.008	0.033
H4b	PP→SAD→PAR	-0.082	3.286	-0.133	-0.033	0.001
H4c	PP→GAD→SAD→PAR	-0.039	3.095	-0.066	-0.017	0.002

PP, Parental Phubbing; GAD, General Anxiety Disorder; SAD, Social Anxiety Disorder; PAR, Physical Activity Rating.

### 5.4 Network analysis

Network analysis, based on the Gaussian graphical model (GGM), constructs networks among variables and provides metrics that describe the associations and structure between observed variables—metrics that are difficult to capture using traditional latent variable models (59, 60). In practical research, these metrics can offer researchers new perspectives on the relationships between variables, thereby helping to address research questions that latent variable models cannot adequately handle. The network reflects cross-sectional conditional associations only and cannot be interpreted as dynamic or causal. The analysis serves two specific purposes: (1) complementing SEM results by identifying central nodes, and (2) generating hypotheses for future longitudinal research.

**Figure 3:**
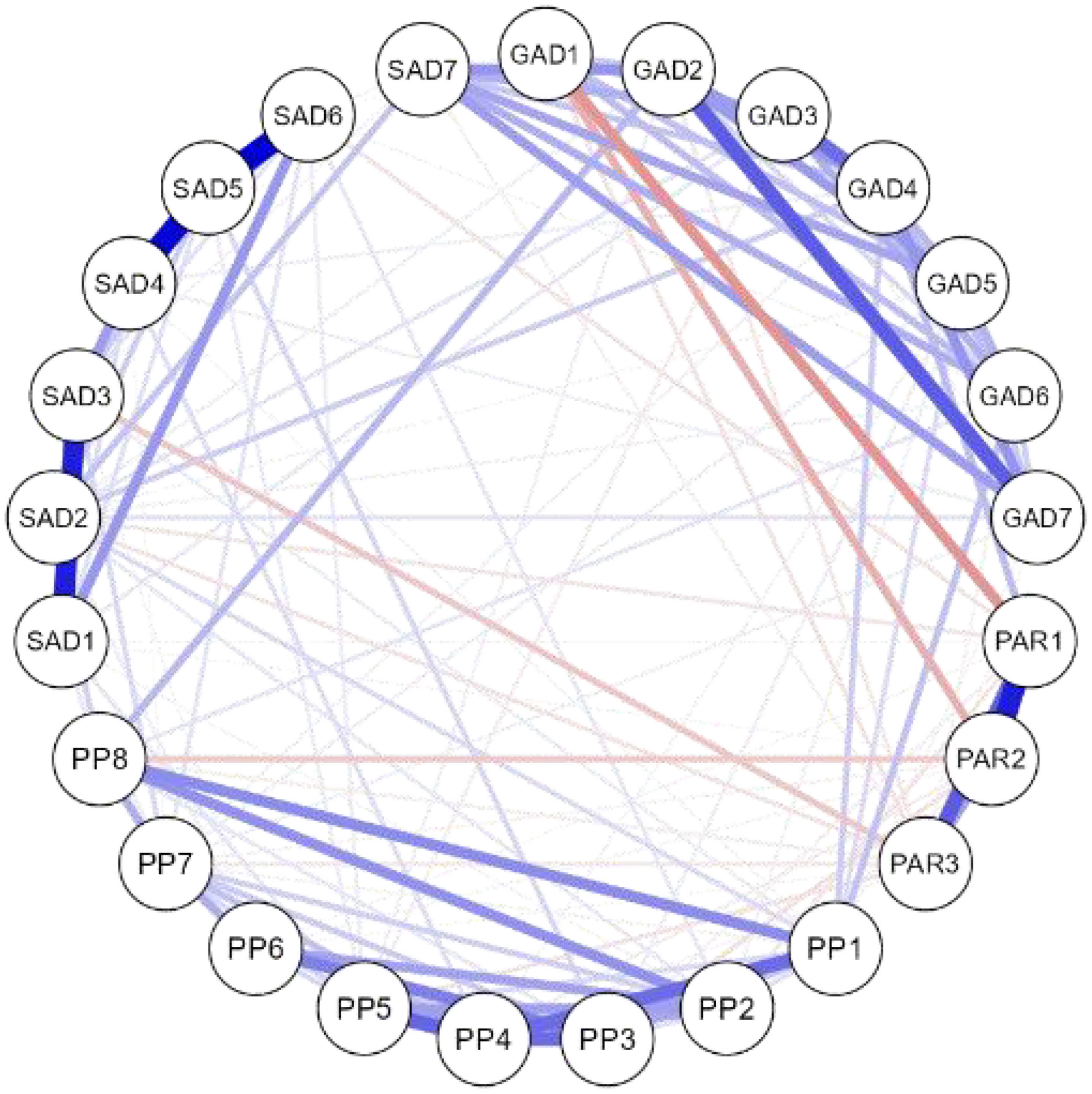
Results of network analysis.

Network analysis was conducted using JASP version. A GGM was estimated using the EBICglasso algorithm (LASSO regularization + EBIC model selection) with a tuning parameter (γ) set to 0.5. Because all items were measured on 5-point Likert scales and treated as ordinal variables, polychoric correlations were used as input. No missing data were present in the final sample (N = 509). Edge weight accuracy was assessed via non-parametric bootstrapping with 2, 500 resamples, generating 95% confidence intervals for each edge. Case-dropping subset bootstrapping was performed with 500 bootstraps, dropping 10% to 50% of cases in 10% increments. The centrality stability (CS) coefficient was computed for each centrality index; a CS coefficient > 0.25 indicates acceptable stability (59). Bootstrap-based centrality difference tests (2, 500 bootstraps) were conducted to compare centrality indices across nodes, with significance set at p < 0.05. **Figure 3** illustrates the connections among the variables. The associations between items are represented by lines, with blue lines indicating positive correlations and red lines indicating negative correlations. Thicker lines represent stronger correlations.

In network analysis, Betweenness (betweenness centrality) reflects the extent to which a node serves as a “bridge”—the higher the value, the stronger its control over information flow, and its removal is statistically associated with reduced network efficiency. Closeness (closeness centrality) measures the average distance between a node and all other nodes, with higher values indicating faster information propagation. Strength represents the sum of a node’s connection weights, where a larger value suggests stronger interaction or resource flow. Expected Influence predicts a node’s impact by combining connection weights and neighbor attributes, with high values implying a significant ability to amplify or suppress network states.

**Figure 4:**
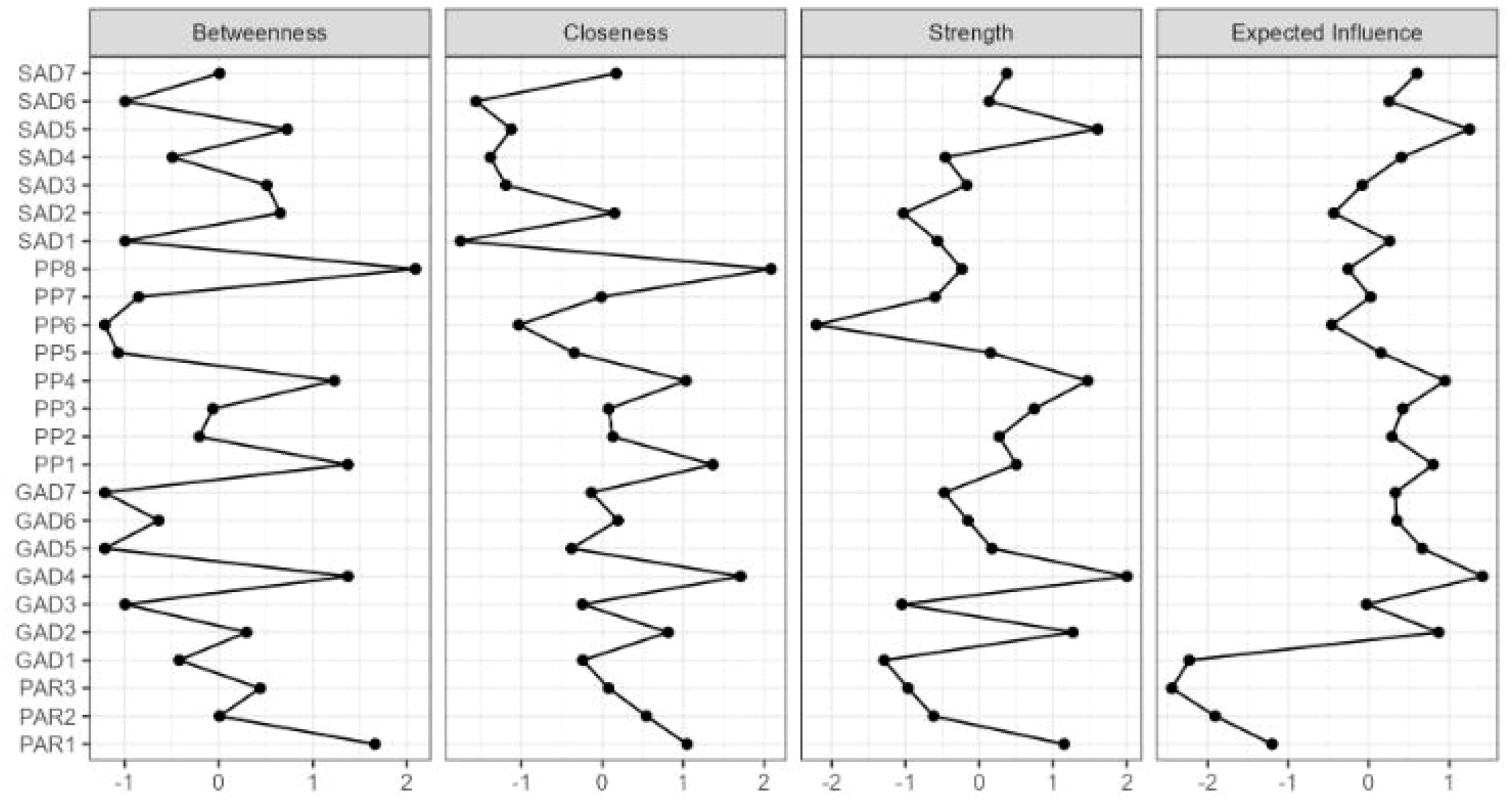
Centrality plot.

**Figure 4** display the distribution of these four metrics across the 25 items. The tabulated data reveal that GAD4 (“In the past week, I found it hard to relax”) scored high on all four metrics—Betweenness (1.372), Closeness (1.71), Strength (1.998), and Expected Influence (1.414)—indicating its role as a core node in the network. It not only functions as a critical bridge but also efficiently disseminates information and exerts a strong positive influence.

In contrast, PP8 (“When I go out with my parents, they play with their phones”) exhibits extremely high Betweenness (2.089) and Closeness (2.088) but negative Strength (-0.237) and Expected Influence (-0.257), suggesting that while it occupies a key pathway, it may suppress network interactions in practice. PAR1 (exercise intensity) shows notable Betweenness (1.659) and Strength (1.15) but negative Expected Influence (-1.201), implying it may transmit inhibitory effects through strong connections. Meanwhile, GAD2, despite moderate Betweenness, has high Closeness (0.814), Strength (1.268), and Expected Influence (0.87), demonstrating efficient information propagation and a positive driving effect.

By comparison, variables such as GAD1 (“In the past week, I felt nervous, anxious, or on edge”), GAD3 (“In the past week, I worried too much about various things”), PP6 (“When I play with my parents, they also use their phones”), and SAD1 (“I feel nervous and afraid when talking face-to-face with others”) generally show low or even negative values across metrics, indicating their peripheral positions in the network and limited influence.

## 6 Discussion

The development of digital technology has made parents’ excessive use of electronic media a significant public issue. Adolescents’ psychological and health problems have also garnered widespread attention. Against this backdrop, this study, based on the SOR model, establishes potential pathways through which parents’ phubbing behavior harms adolescents’ mental and physical health. The findings indicate that parental phubbing is significantly positively associated with adolescents’ anxiety levels, including generalized anxiety and social anxiety, while also reducing their physical activity levels. This suggests that, for adolescents’ development, parents’ phubbing behavior greatly damages their mental and physical health, exerting an influence that cannot be overlooked.

The results show that the frequency of parental phubbing is significantly positively associated with adolescents’ generalized anxiety and social anxiety, which is largely consistent with conclusions from related studies. When parents are engrossed in their phones, adolescents perceive this as a form of neglect and rejection, leading to negative emotions (61). The interruption in communication caused by phubbing reduces the quality of parent-child interactions, fostering a tendency toward social avoidance in adolescents (11). Parents also serve as role models for their children. Although some parents believe that merely being physically present with adolescents fulfills their parenting role, adolescents are like mirrors of their parents—their behavior reflects that of their parents (62). When parents reduce communication and conversation with adolescents, the latter also subconsciously decrease their social interactions with the outside world (63). The diminished frequency and duration of family interactions due to excessive phone use not only contribute to generalized anxiety but also trigger social anxiety (64).

What is particularly concerning is that parents’ phubbing behavior also significantly negatively associated with the frequency and quality of adolescents’ physical activities. A possible explanation is that adolescents may imitate their parents’ excessive use of electronic devices, leading them to prefer sedentary activities (such as scrolling through phones or playing video games) over engaging in physical exercise (65). From a time allocation perspective, if parents spend more time on electronic devices, they inevitably dedicate less time to organizing or accompanying their children in outdoor activities, indirectly limiting adolescents’ opportunities for exercise. Physical activity is a crucial health-related factor, and parental involvement in adolescents’ sports participation is considered one of the most significant positive correlates, making it worth parents’ investment of more time (66). Parental engagement in adolescents’ physical activities not only positively impacts weight management but also exerts a profound influence on their mental well-being (67).

From an indirect perspective, generalized anxiety and social anxiety exhibit significant indirect associations in the relationship between parental phubbing and physical activity between parental phubbing behavior and adolescents’ physical activity levels. This is not only because parental phubbing increases the likelihood of adolescents developing generalized and social anxiety, but also because heightened anxiety further raises the probability of sports avoidance. This suggests that parental phubbing can directly reduce adolescents’ participation in physical activities while also indirectly suppressing their motivation to exercise by exacerbating anxiety. Previous research indicates that family factors shape children’s physical and mental health. A positive correlation exists between family sports culture and adolescents’ engagement in physical activities (32). In families that emphasize athletic development, children may receive more encouragement and are more likely to exercise independently (68). However, when parents are overly absorbed in digital devices (i.e., phubbing), it not only weakens the family’s sports culture but may be statistically linked to adolescents’ psychological vulnerability through emotional neglect. Studies suggest that parental phubbing reduces parent-child interaction frequency and diminishes children’s perceived emotional support (11).

The ENA results further reveal significant differences in the nodal characteristics of the generalized anxiety and social anxiety dimensions in terms of network structure and functional influence. The study found that core nodes in the generalized anxiety dimension exhibit high betweenness centrality and positive expected influence, a pattern that may reflect the hub-like role of cognitive symptoms such as excessive worry within the network. Their structural dominance stems from their ability to simultaneously activate multiple interrelated anxiety symptoms. In contrast, certain nodes in the social anxiety dimension, while possessing high betweenness centrality, demonstrate negative functional influence. This paradoxical pattern may reflect the inhibitory effect of social avoidance behaviors on symptom propagation. Although topologically central, these nodes may functionally restrict rather than facilitate symptom diffusion, manifesting as social withdrawal (69). In particular, nodes related to family interactions display unique inhibitory regulatory characteristics—combining strong connection strength with negative influence—suggesting that family environmental factors may serve as a buffer against anxiety symptoms (70). This implies that while family-related nodes are well-integrated into the anxiety network, they may exert a protective effect by dampening symptom escalation.

## 7 Conclusion

This study, grounded in the SOR theoretical model, systematically examined the statistical associations linking parental phubbing to adolescents’ psychological and physical health outcomes. The findings reveal that parental phubbing is positively associated with adolescents’ generalized anxiety and social anxiety, and negatively associated with their participation in physical activities.

Theoretical contributions. These findings extend the SOR model in three ways. First, previous applications of the SOR model to family technology use have typically treated internal states as a single psychological construct. This study demonstrates that distinguishing between generalized anxiety and social anxiety reveals different patterns of association with parental phubbing. Second, the network analysis results suggest that generalized anxiety functions as a central hub node within the symptom network, whereas social anxiety exhibits a distinct suppressive pattern. This implies that not all anxiety symptoms play the same role in translating family environment stimuli into behavioral outcomes. Third, the chain mediation findings suggest that generalized anxiety and social anxiety may operate sequentially. Parental phubbing may first trigger generalized worry about daily life, which then heightens concerns about social evaluation, ultimately contributing to avoidance of physical activity. This sequential pathway has not been previously documented in the parental phubbing literature.

Practical implications. The psychological and physical well-being of adolescents is a critical priority for their future development. The correlational findings suggest that parents may wish to be mindful of the modeling effects of their own device use on their children. Maintaining moderate digital engagement in family life and increasing face to face parent child interactions may promote adolescents’ mental health and physical activity. Such practices may help alleviate adolescents’ anxiety symptoms and create a more supportive family environment for their healthy growth.

Unexpected findings. One finding was not anticipated by our original hypotheses. The network analysis revealed that certain social anxiety nodes exhibited negative expected influence within the network. This suppressive pattern was initially inconsistent with our hypothesis that both types of anxiety would be positively associated with physical activity avoidance. A possible explanation is that social anxiety leads to social withdrawal, which may coincidentally reduce exposure to team-based sports where peer evaluation is prominent. This paradoxical effect suggests that different anxiety types may have different functional consequences. Future research should replicate this unexpected pattern in independent samples.

It is important to note, however, that the explanatory power of the model for physical activity and social anxiety was weak-to-moderate by conventional standards. This suggests that while parental phubbing is significantly associated with these outcomes, other unmeasured factors—likely play substantial roles. Thus, the present findings should be interpreted as evidence of correlational associations rather than comprehensive explanation.

## 8 Limitations and future research

First, regarding sample and cultural context. The sample was drawn exclusively from three schools in Shaanxi Province, China. This regional and cultural specificity limits the generalizability of our findings to other populations. Parent child interaction patterns and norms regarding technology use may differ across cultures. For example, parental phubbing might carry different meanings or consequences in collectivist versus individualist societies. Future research should include cross cultural samples to test whether the observed associations replicate in diverse cultural contexts.

Second, regarding measurement and design. This study relied on self-report measures, which are susceptible to memory bias and social desirability effects. The cross sectional design precludes causal inferences. All reported associations should be interpreted as statistical correlations rather than causal effects. We also acknowledge that potential confounding variables such as school environment and peer relationships were not fully controlled. Future research should employ longitudinal designs, objective measurements (e.g., device monitored screen time, accelerometer recorded physical activity), and include additional covariates to strengthen causal claims.

Third, regarding intervention recommendations. Rather than offering general advice, we suggest specific actionable measures. Parents could establish designated family device free time, such as during meals or the first hour after returning home. Another concrete strategy is the “phone stacking” practice, where all family members place their phones in a common basket during shared activities. Schools could implement parent education workshops that teach these specific techniques. Future research should evaluate the effectiveness of such targeted interventions using randomized controlled designs to establish causal effects on adolescent anxiety and physical activity.

## Data Availability

The raw data supporting the conclusions of this article will be made available by the authors, without undue reservation.
